# Epigenetic activation of the elongator complex sensitizes gallbladder cancer to gemcitabine therapy

**DOI:** 10.1186/s13046-021-02186-0

**Published:** 2021-11-25

**Authors:** Sunwang Xu, Cen Jiang, Ruirong Lin, Xiaopeng Wang, Xiaoqiang Hu, Wei Chen, Xiangjin Chen, Tao Chen

**Affiliations:** 1 Department of Biliary-Pancreatic Surgery, Renji Hospital, School of Medicine, Shanghai Jiao Tong University, Shanghai, 200127 China; 2 Department of Thyroid and Breast Surgery, The First Affiliated Hospital of Fujian Medical University, Fuzhou, 350005 China; 3 Central Laboratory, Fujian Medical University Union Hospital, Fuzhou, 350001 China

**Keywords:** Gallbladder cancer, Elongator complex, DNA methylation, Gemcitabine, Decitabine

## Abstract

**Background:**

Gallbladder cancer (GBC) is known for its high malignancy and multidrug resistance. Previously, we uncovered that impaired integrity and stability of the elongator complex leads to GBC chemotherapy resistance, but whether its restoration can be an efficient therapeutic strategy for GBC remains unknown.

**Methods:**

RT-qPCR, MS-qPCR and ChIP-qPCR were used to evaluate the direct association between ELP5 transcription and DNA methylation in tumour and non-tumour tissues of GBC. EMSA, chromatin accessibility assays, and luciferase assays were utilized to analysis the DNA methylation in interfering PAX5-DNA interactions. The functional experiments in vitro and in vivo were performed to investigate the effects of DNA demethylating agent decitabine (DAC) on the transcription activation of elongator complex and the enhanced sensitivity of gemcitabine in GBC cells. Tissue microarray contains GBC tumour tissues was used to evaluate the association between the expression of ELP5, DNMT3A and PAX5.

**Results:**

We demonstrated that transcriptional repression of ELP5 in GBC was highly correlated with hypermethylation of the promoter. Mechanistically, epigenetic analysis revealed that DNA methyltransferase DNMT3A-catalysed hypermethylation blocked transcription factor PAX5 activation of ELP5 by disrupting PAX5-DNA interaction, resulting in repressed ELP5 transcription. Pharmacologically, the DNA demethylating agent DAC eliminated the hypermethylated CpG dinucleotides in the ELP5 promoter and then facilitated PAX5 binding and reactivated ELP5 transcription, leading to the enhanced function of the elongator complex. To target this mechanism, we employed a sequential combination therapy of DAC and gemcitabine to sensitize GBC cells to gemcitabine-therapy through epigenetic activation of the elongator complex.

**Conclusions:**

Our findings suggest that ELP5 expression in GBC is controlled by DNA methylation-sensitive induction of PAX5. The sequential combination therapy of DAC and gemcitabine could be an efficient therapeutic strategy to overcome chemotherapy resistance in GBC.

**Supplementary Information:**

The online version contains supplementary material available at 10.1186/s13046-021-02186-0.

## Background

Gallbladder cancer (GBC) is a rare but highly malignant tumour with a dismal prognosis and a 5-year survival rate of less than 5% in locally advanced or metastatic stages [1]. The clinical outcomes of GBC patients remain unsatisfactory mainly because of multidrug resistance. Gemcitabine-based chemotherapy is the first-line treatment for locally advanced or metastatic GBC, but a poor response commonly occurs [2, 3]. One of the major mechanisms of gemcitabine resistance is failure of gemcitabine-induced apoptosis to cause cytotoxic effects [4]. Previously, we revealed a gemcitabine resistance mechanism in GBC cells: impaired integrity and stability of the elongator complex disrupts internal ribosomal entry site (IRES)-driven p53 translation and accumulation and reduces p53-mediated apoptosis under gemcitabine treatment [5]. Whether restoring the integrity and stability of the elongator complex could be an efficient therapeutic strategy to sensitize GBC cells to gemcitabine therapy is worth further exploration.

The elongator complex is organized by an ELP456 subcomplex possessing a hexameric RecA-like ATPase that hydrolyses ATP and provides tRNA-specific binding sites and an ELP123 subcomplex containing 5-carbamoylmethyluridine (cm^5^U) catalytic activity and catalysing uridine into cm^5^U [6]. The elongator complex controls mRNA translation by catalysing tRNA modifications at the wobble uridine (uridine 34, U34) to drive tumour initiation, progression, metastasis, and targeted therapy resistance [7–9]. ELP5 is a core subunit of the elongator complex. In our previous study, we revealed that ELP5 is essential for maintaining the integrity and stability of the elongator complex to induce gemcitabine cytotoxic effects in GBC cells [5]. Although we have uncovered that ELP5 acts as a tumour suppressor affecting gemcitabine therapeutic responses and survival outcomes in GBC patients [5], the mechanism inducing ELP5 repression in GBC patients remains unclear.

Accumulating evidence has provided an association between epigenetic changes, especially DNA methylation, and the transcription of genes in tumours [10–12]. The methylation of cytosine (5-methylcytosine, 5mC) in CpG dinucleotides can repress gene transcription and thus silence gene function; in contrast, eliminating methylation on CpGs can reactive transcription. DNA demethylating agents, such as 5-aza-2′-deoxycytidine (decitabine, DAC) and 5-azacytidine, have been used to reactivate silenced tumour suppressor transcription and synergize with traditional chemotherapies [13]. For example, epigenetic activation of hypermethylated OCT2 by DAC was found to activate OCT2 expression to enhance oxaliplatin therapy in renal cell carcinoma [14], and DAC was found to activate STAT3 signalling pathways with a DNA demethylation approach to improve cisplatin efficacy in basal-like bladder cancer [15]. However, whether DNA demethylating agents can be efficient in synergizing with gemcitabine cytotoxic effects in GBC is unknown.

Here, we found evidence to support a transcriptionally repressed status of ELP5 in GBC tissues as the molecular mechanism by which DNMT3A-mediated DNA hypermethylation blocks PAX5-induced activation of ELP5. We also revealed that the demethylating agent DAC could restore the U34 tRNA catalytic function of the elongator complex by reactivating ELP5 expression. More importantly, we generated a sequential combination therapy in which DAC was used to sensitize GBC cells to gemcitabine, and we propose that DNA demethylating agents can serve as a therapeutic strategy to overcome chemotherapy resistance in GBC.

## Materials and methods

### Clinical samples

All GBC tissues were obtained from Renji Hospital affiliated to Shanghai Jiao Tong University School of Medicine between January 2010 and December 2018 with the patients’ consent. All patients enrolled in this study were underwent radical cholecystectomy, diagnosed GBC by pathology, and did not receive any radiotherapy or chemotherapy before surgery. This study was reviewed and approved by the Ethics Committees of Renji Hospital affiliated to Shanghai Jiao Tong University School of Medicine, and the written informed consent was obtained from all subjects in this study. All the research was carried out in accordance with the provisions of the Helsinki Declaration of 1975.

### Cell culture and reagents

NOZ, GBC-SD, and human embryonic kidney 293 T (HEK293T) cells were purchased from the Health Science Research Resources Bank (Osaka, Japan), the Cell Bank of Type Culture Collection of Chinese Academy of Science (China), and the American Type Culture Collection (American), respectively. NOZ cells were cultured in William’s E medium (Hyclone), GBC-SD and HEK293T cells were cultured in Dulbecco’s modified Eagle’s medium (Hyclone). All cell lines were supplemented with 10% fetal bovine serum (Gibco) and penicillin-streptomycin (Hyclone), incubated in a humidified chamber with 5% CO_2_ at 37 °C, and ensured to be mycoplasma-negative cultures by monthly mycoplasma testes. Gemcitabine (GEMZAR) was purchased from Eli Lilly (American), demcitabine and puromycin were purchased from MedChem Express (American).

### Stable knockdown cell lines construction

Recombinant lentivirus delivering short hairpin RNAs (shRNAs) were produced in HEK293T cells and the viruses were harvest and used to infect NOZ and GBC-SD cells. Then, the cells were selected by puromycin to eliminate uninfected cells to generate stable cell line. The shRNAs targeting PAX5, DNMT3A, DNMT3B, and DNMT1 were obtain from Biochemistry and Molecular Cell Biology, Shanghai Jiao Tong University School of Medicine. The sense sequence of shRNAs were: shPAX5_1: 5′-GTGATGTAGACAATAATTA-3′, shPAX5_2: 5′-CGGACCAGCAGGACAGGAC-3′, shDNMT3A: 5′-CCCAAGGTCAAGGAGATTA-3′, shDNMT3B: 5′-GCCCATTTGACTTGGTGAT-3′, and shDNMT1: 5′-TGGACGACCCTGACCTCAA-3′.

### CRISPR/Cas9-mediated mutagenesis

To achieve the genomic deletion of PAX5 binding site (ΔPAX5BS) on ELP5 promoter by CRISPR/Cas9-mediated mutagenesis, the optimal sgRNA sequence (5′-CGCAGAGCGCTGGGCCGGAG-3′) were cloned into lentiCRISPR-V2 vector (Addgene). The resulting plasmid together with psPAX and pMD2.G plasmids were introduced into HEK293T cells to generate lentivirus using standard procedures. And then the NOZ cells were infected and selected with puromycin, and the puromycin-resistant single cell clones were isolated by limiting dilution. Upon clone expansion, PAX5 binding site deleted clones were assessed by PCR and the following Sanger sequencing. Primers for PCR were: ΔPAX5BS-PCR-F: 5′-AGCGGCGCGCAAAGGGCCGC-3′ and ΔPAX5BS-PCR-R: 5′-GGACGGTAAAATGGCGCCTG-3′.

### Cell viability assays

Cells in single-cell suspension were plated at 4000 cells in 100 μl of culture medium per well of 96-well plates. 72 h after chemical reagents treatment, 10 μl of Cell Counting Kit-8 (Dojindo) solution was added to cells directly, and then incubated at 37 °C for 2 h, followed by measurement of the absorbance at 450 nm using a Synergy 2 micro-plate reader (Biotek). The relative cell viability was calculated and normalized to the vehicle treated group.

### RNA extraction and real-time quantitative PCR (RT-qPCR)

Total RNA was extracted from cells using TRI Reagent (Sigma-Aldrich) following the manufacturer’s introductions. 1 μg of total RNA was reverse transcribed using the 1st Strand cDNA Synthesis SuperMix (Yeasen) into cDNA. qPCR was performed by qPCR SYBR Green Master Mix (Yeasen) in triplicate using the Applied Biosystems ViiA TM 7 Real-Time PCR system (Applied Biosystems). The Ct values obtained from different samples were compared using the 2 − ΔΔCt method, and ACTB served as an internal reference gene. The primers were used for RT-qPCR as follows: ELP5 (F: 5′-AGCGAGGAAGAGTTTCGTGA-3′, R: 5′-GGAAAGGCCTCCTCAGTTTT-3′), ACTB (F: 5′-CATGTACGTTGCTATCCAGGC-3′, R: 5′-CTCCTTAATGTCACGCACGAT-3′).

### DNA copy number assessment

The exact copy numbers of ELP5 transcripts per cell in the GBC tissues were quantified by using RT-qPCR assays. In this assay, serially diluted pcDNA3.0-ELP5-expressing plasmids were used as templates to formulate standard curves, and then the exact copies of ELP5 in GBC tissues were calculated accordingly.

### Chromatin immunoprecipitation (ChIP)-qPCR assays

The ChIP assays were performed by using a SimpleChIP Enzymatic Chromatin IP Kit (Cell Signaling Tech) following the manufacturer’s introduction. The immunoprecipitified DNA were quantified by qPCR method. The primers were used for ChIP-qPCR as follows: ELP5 (F: 5′-AAGGAGCAGGGAAGGAGGGG-3′, R: 5′-CTAAAGGACCCCCGAGCTC-3′). Antibody used for ChIP against PAX5 (#8970) were purchased from Cell Signaling Tech, and 5mC (SAB2702243) was purchased from Sigma-Aldrich.

### Bisulfite sequencing PCR (BSP) and methylation-specific quantitative PCR (MS-qPCR) assays

Genomic DNA was extracted from cells or tissues by using the QIAamp DNA blood mini kit (Qiagen), and bisulfite treatment was performed by using the DNA Methylation-Gold Kit (Zymo Research), following the manufacturer’s introductions. For BSP assays, modified DNA was amplified and PCR products were gel-purified and sub-cloned into a pESI-T vector system (Yeasen). Ten colonies were sequenced to assess the degree of methylation and each CpG site by QUMA [16]. For MS-qPCR assays, the modified DNA was amplified to determine the methylation status of the promoter region of target gene as described previously [17]. The primers were used for BSP as follow: ELP5 (F: 5′-GGGGGGAAAGTAGAGAGTGGTT-3′, R: 5′-ACCCAACTACAAAAACTACAACCC-3′), and for MS-qPCR as follow: ELP5 (F: 5′-GGGAAAGTAGAGAGTGGTTCGT-3′, R: 5′-AATCCTTTAAACGATAAAATAACGC-3′) and ACTB (F: 5′-TGGTGATGGAGGAGGTTTAGTAAGT-3′, R: 5′-AACCAATAAAACCTACTCCTCCCTTAA - 3′).

### Western blot assays

Western blot was performed using standard procedures. Cell lysates were lysated by radioimmunoprecipitation lysis buffer containing 0.1% sodium dodecyl sulphate (SDS) and containing proteinase inhibitor, and quantified with the Micro BCA Protein Assay Kit (Thermo Fisher Scientific). Aliquots of 20 μg of protein were electrophoresed through 10% SDS polyacrylamide gels and were then transferred to polyvinyl difluoride membranes (Millipore), followed by blocking in 5% skim milk at room temperature for 1 h and then incubation with primary antibodies at 4 °C overnight. Secondary antibodies were labeled with horseradish peroxidase, and the signals were detected using the ECL Kit (Millipore). The images were analyzed using ImageJ 1.43 software. β-actin served as an internal control for the whole-cell lysates. Antibody against ELP5 (sc-514,018, dilution 1:100) was purchased from Santa Cruz, DNMT3A (#32578, dilution 1:1000) and PAX5 (#8970, dilution 1:1000) were purchased from Cell Signaling Tech, ELP3 (ab190907, dilution 1:3000) and ELP4 (ab133687, dilution 1:1000) were purchased from Abcam, Flag (SAB1306078, dilution 1:10000) and β-actin (A1978, dilution 1:10000) were purchased from Sigma-Aldrich.

### Luciferase assays

For Dual-luciferase reporter assay, HEK293T cells were seed in 12-well plate at a density of 2 × 10^5^ cells per well and incubated overnight. pGL3-Basic, each ELP5 promoter sequences contained pGL3-based constructs, and pRL-Amp were co-transfected by using Lipofectamine 2000 (Invitrogen) according to the manufacturer’s protocol. Forty-eight hours after transfection, cells were harvested, lysed and Fluc and Rluc activities were determined according to the manufacturer’s protocol of Dual-Luciferase Reporter Assay System (Promega). The ELP5 promoter activity was calculated by the ratio of Fluc to Rluc.

### In vitro methylation

For in vitro methylation, reporter plasmids of pGL3 vector were methylated by M.SssI (New England Biolabs) or mock methylated in the absence of M.SssI and SAM. The efficiency of in vitro methylation was detected by HpaII (New England Biolabs) digestion that the totally methylated reporter plasmids would not be digested into small fragments by HpaII. After that, the in vitro methylated reporter plasmids were transfected to HEK293T cells and perform dual-luciferase reporter assays.

### Northern blot assays

Total RNA was extracted and then 10 μg of total RNA was electrophoresed through 10% polyacrylamide gels containing 0.5 × TBE, 7 m urea and 50 μg/ml [(N-acryloy-lamino)phenyl]mercuric chloride, and transfer to nylon membrane and probed with oligonucleotide probe labeled with digoxin followed the protocol of previously described [18]. The probe sequences for tE UUC was 5′-TTCCCATACCGGGAGTCGAACCCG-3′.

### Electrophoretic mobility shift assay (EMSA)

EMSA was performed by using a LightShift Chemiluminescent EMSA Kit (Thermo Fisher Scientific) following the protocol of previously described [19]. Briefly, 5′-Biotin-labeled, single-stranded oligonucleotides used for PAX5/DNA binding assay were synthesized and annealed. Equal amounts of immunopurified Flag-PAX5 protein was incubated in a 20 μl reaction mix containing 1 ng biotin-labeled annealed oligonucleotide at room temperature for 20 min. Protein/DNA complexes were electrophoresed through 6% DNA retardation gel in 0.5 × TBE and transferred to nylon membrane. After UV-light crosslinking and blocking, the membrane was incubated in Stabilized Streptavidin-Horseradish Peroxidase Conjugate buffer and substrate buffer, and the signalling was exposed and recorded via ChemiDoc XRS+ (Bio-Rad) equipped CCD camera. Sequences of single-stranded oligonucleotides for the methylated CpG probe were: 5′-biotin-AGAGCGCTGGGCmCGGAGCGGCCTCC-3′ and 3′-TCTCGCGACCCGGCmCTCGCCGGAGG- biotin-5′, for the unmethylated probe were 5′-biotin-AGAGCGCTGGGCCGGAGCGGCCTCC-3′ and 3′-TCTCGCGACCCGGCCTCGCCGGAGG-biotin-5′.

### Chromatin accessibility assay

The chromatin accessibility assay was performed by chromatin accessibility by real-time PCR (CHART-PCR) method as previously described [20]. Cell pellets were resuspended in ice-cold Nonidet P-40 lysis buffer (10 mM Tris (pH 7.4), 10 mM Nacl, 3 mM MgCl_2_, 0.15 mM spermine, 0.5 mM spermidine, and 0.5% Nonidet P-40) and incubated on ice for 5 min. The suspension was centrifuged at 3000 rpm for 5 min to pellet the nuclei. And then, the nuclei were suspended and digested with 5 units of micrococcal nuclease, MNase (New England BioLabs) or mock for 15 min. Purified genomic DNA was subjected to qPCR and the relative level of MNase resistance was calculated after normalization to mock-digested DNA. Primers for qPCR are following: PAX5BS (F: 5′-AAGGAGCAGGGAAGGAGGGGGAGG-3′, R: 5′-AGCGGCTAAAGGACCCCCGAGCTCGG-3′), PAX5BS-100 bp (F: 5′-CTGCGCAGGCGCGCTAGGGGGCTGC-3′, R: 5′-CAGGCCCGTTCTCCGCTGCCG-3′), and PAX5BS + 100 bp (F: 5′-GCAGCCTCTGCAGCTGGGTTTCCC-3′, R: 5′-GGGAAGAGGAGGGGGAAAGACAGGA-3′).

### Xenograft model

For the xenograft experiments, 4-week-old male BALB/c athymic nude mice were housed in laminar flow cabinets under specific pathogen-free conditions with food and water provided ad libitum. In all, 1 × 10^6^ NOZ or 2 × 10^6^ GBC-SD cells in 100 μl of PBS were injected subcutaneously into the right axilla of each mouse to establish the GBC xenograft model. When the mean tumour sizes reached 100 mm3, mice were randomized to receive vehicle, DAC (5 mg/kg), or GEM alone (50 mg/kg, twice a week) or in sequential combination at indicated time points. The length and width of the tumors (in mm) were measured with calipers before vehicle or gemcitabine injection. The tumour volume was calculated using the formula (length × width^2^)/2. All the mice were killed at the end of the indicated intraperitoneal injection, and subcutaneous tumors were collected and weighed. The tumour volume and weight were presented as the means ± S.D. (*n* = 5–8). In vivo studies were conducted in accordance with the National Institutes of Health Guidelines for the Care and Use of Laboratory Animals, and the study procedures were approved by the Institutional Animal Care and Use Committee of Renji Hospital affiliated to Shanghai Jiao Tong University School of Medicine.

### Immunohistochemistry (IHC) analysis

The tissue slides were deparaffinized, treated with 3% H_2_O_2_ for 10 min, autoclaved in 10 mM citric sodium (pH 6.0) for 30 min to unmask antigens, rinsed in phosphate-buffered saline and then incubated with primary antibodies at 4 °C overnight, followed by incubation with biotinylated secondary antibody for 1 h at room temperature. Signal amplification and detection were performed using the DAB system according to the manufacturer’s instructions, and the stained sections were photographed and converted to a digital image at 100 × and 400 × under a light microscope equipped with a camera (Olympus). The intensity score was determined by evaluating staining intensity of positive staining (0 = none; 1 = weak; 2 = moderate; 3 = strong). The proportion score representing the percentage of positively stained cell (0 = none; 1 = 1–10%; 2 = 11–50%; 3 = 51–80%; 4 = 81–100%). The overall protein expression in each sample was expressed as histoscore, which was multiplication product of the intensity score (0–3) and proportion score (0–4) and is between 0 and 12, with a maximum of 12. Sample with histoscore of more than four were considered to be high, and less than four were considered to be low. The staining score was evaluated by two independent pathologists. Antibody against ELP5 (HPA023279, dilution 1:200), DNMT3A (HPA026588, dilution 1:200) and PAX5 (HPA056394, dilution 1:100) was purchased from Sigma-Aldrich.

### Statistical analysis

Data were presented as the means ± S.D. One-Sample Kolmogorov-Smirnov test was applied for normally distributed data examination. Two tailed-unpaired Student’s t-test was applied to compare the difference between two groups, and one-way ANOVA test was applied to compare the difference among three or more groups. Pearson correlation coefficient were used to analysed the correlation of histoscore in IHC staining. All statistical calculation was performed using SPSS software package (version 23.0, IBM SPSS), and a *P* < 0.05 was considered to be statistically significant.

## Results

### Hypermethylation of the ELP5 promoter represses ELP5 expression in GBC

**Fig. 1:**
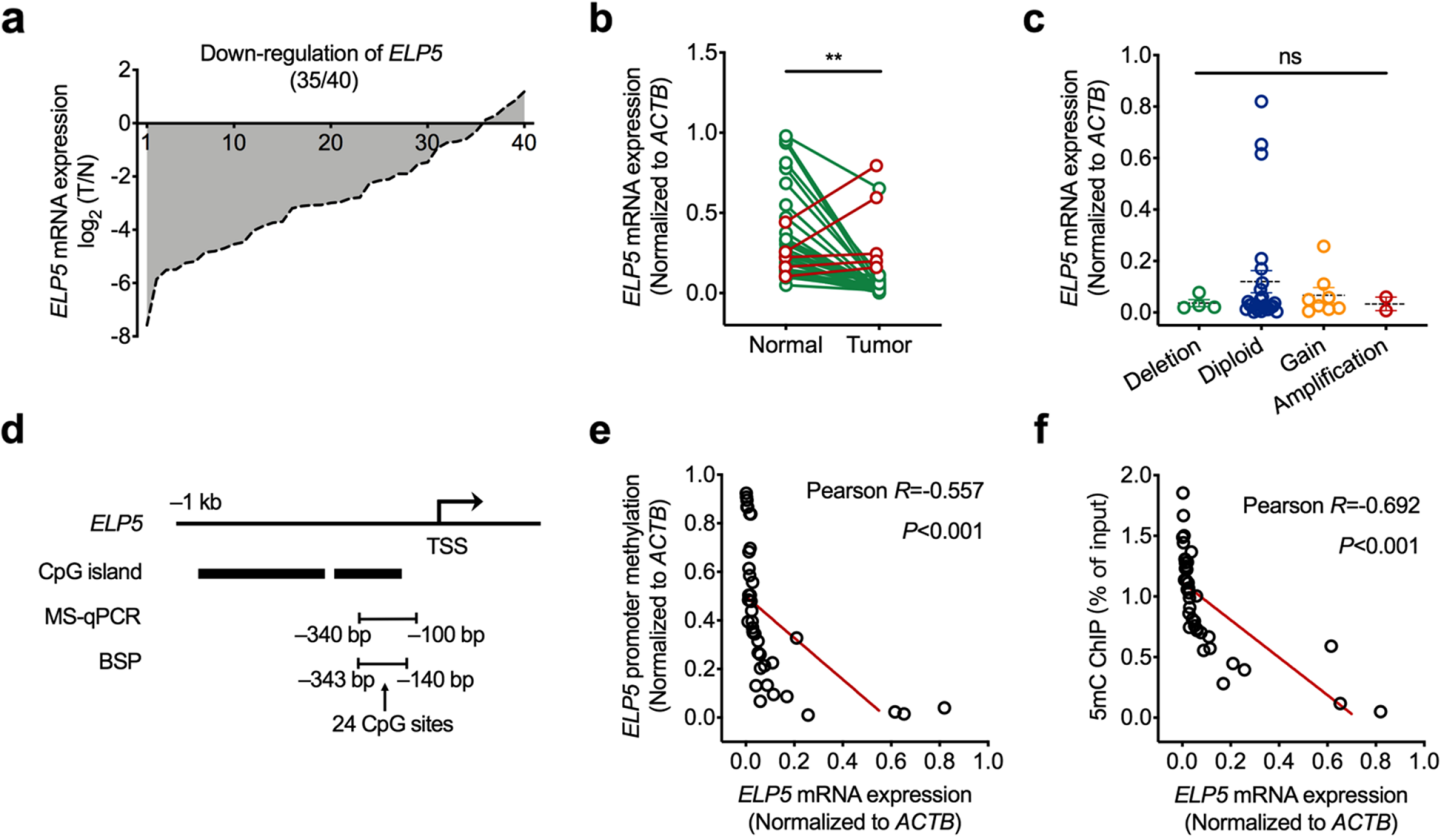
DNA hypermethylation represses ELP5 expression in GBC. **a** Relative expression of ELP5 in GBC tumorous tissues normalized to matched normal tissues. **b** Statistical analysis of ELP5 expression in GBC tumorous tissues and matched normal tissues. Paired student’s t test for statistical analysis, **P < 0.01. **c** Statistical analysis of ELP5 expression in GBC tissues with different DNA copy numbers. One-way ANOVA test for statistical analysis, ns, not significant. **d** Schematic diagram of CpG islands on ELP5 promoter region around transcription start site (TSS). **e** Correlation analysis between the promoter methylation levels and transcription levels of ELP5 in GBC tissues, detected by methylation-specific quantitative PCR (MS-qPCR) and real-time quantitative PCR (RT-qPCR), respectively. **f** Correlation analysis between the contents of 5mC in promoter and transcription levels of ELP5 in GBC tissues, detected by anti-5mC chromatin immunoprecipitation (ChIP)-qPCR and MS-qPCR, respectively

To clarify the expression status of ELP5 in GBC tissues, we detected ELP5 mRNA level in 40 GBC tumour tissues and paired adjacent normal tissues. The results showed that ELP5 was drastically downregulated at the mRNA level in tumour tissues in comparison with adjacent normal tissue counterparts in 87.5% of the GBC samples (Fig. 1a, b). However, we did not observe a correlation between ELP5 mRNA level and ELP5 DNA copy number changes (Fig. 1c), suggesting that GBC exhibits a repressed status of ELP5 expression that does not result from DNA copy number alteration.

Promoter hypermethylation is one of the major mechanisms inducing gene transcription repression and silencing. We explored the correlation between promoter methylation and ELP5 silencing in GBC. First, we searched for potential CpG islands within the promoter region of ELP5 through the MethPrimer website [21]. Two CpG islands were predicted upstream of the transcription start site (TSS) within the ELP5 locus (Fig. 1d). Next, we used methylation-specific quantitative PCR (MS-qPCR) to evaluate the relative methylation level of the ELP5 promoter in 40 GBC tumour tissues. The results showed that the relative ELP5 promoter methylation rate was negatively correlated with the ELP5 mRNA level (Pearson *R* = -0.557, *P* < 0.001) (Fig. 1e). The chromatin immunoprecipitation (ChIP)-qPCR assay with 5mC antibody pulldown further confirmed that the content of 5mC in the ELP5 promoter was also negatively correlated with the ELP5 mRNA level (Pearson *R* = -0.692, *P* < 0.001) (Fig. 1f). Taken together, these data reveal that ELP5 is downregulated in GBC tissues and that repression of ELP5 is highly associated with promoter hypermethylation.

### DNMT3A mediates hypermethylation of the ELP5 promoter

**Fig. 2:**
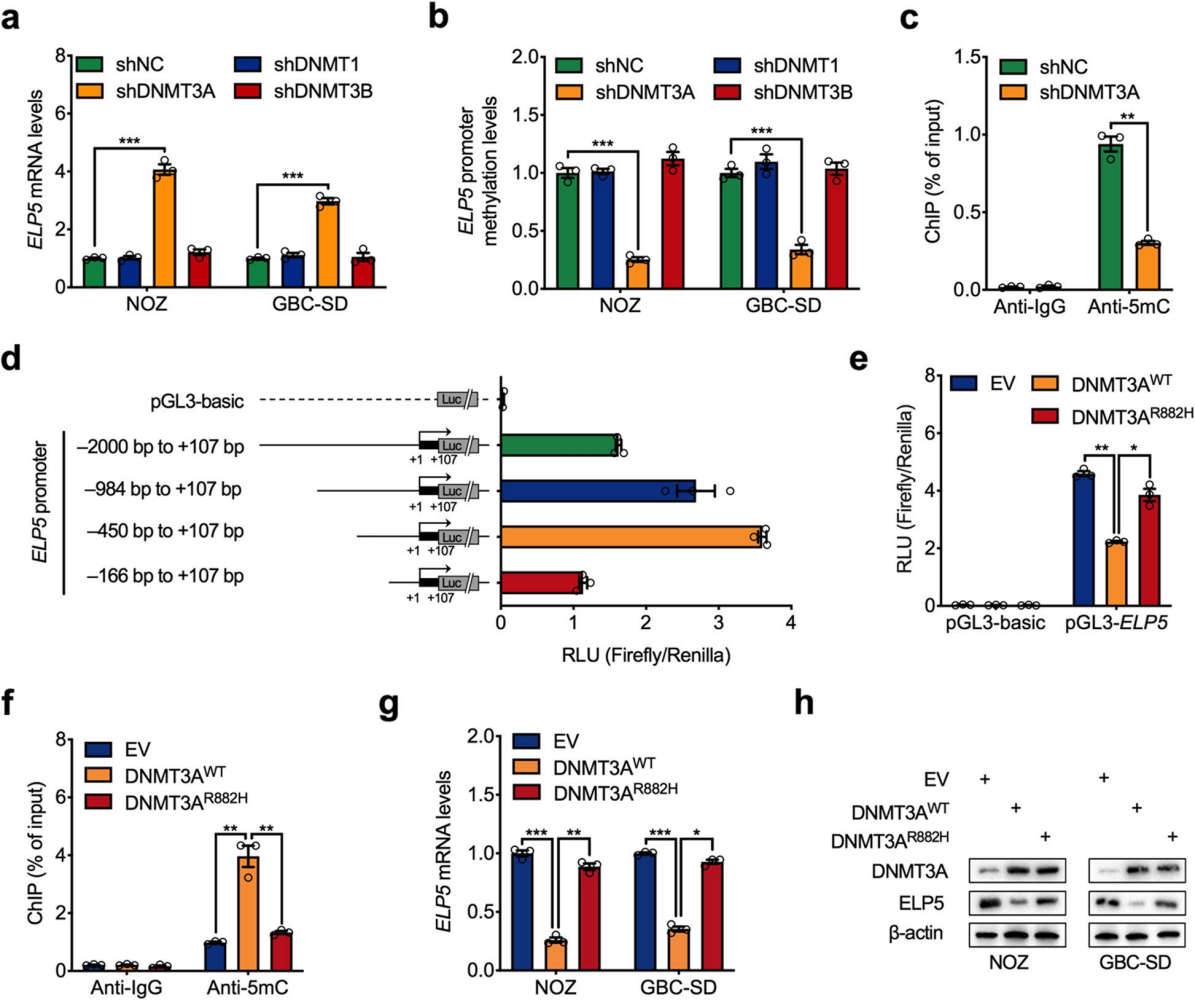
DNMT3A mediates hypermethylation of ELP5. **a** RT-qPCR analysis of ELP5 transcription levels upon DNMT1, DNMT3a, and DNMT3B stably knockdown in NOZ and GBC-SD cells. **b** MS-qPCR analysis of ELP5 promoter methylation levels upon DNMT1, DNMT3A, and DNMT3B stably knockdown in NOZ and GBC-SD cells. **c** ChIP-qPCR analysis of 5mC contents in ELP5 promoter with or without DNMT3A stably silenced in NOZ cells. **d** Luciferase assay to screen the ELP5 promoter region with the highest pro-transcription activity by transfected with a group of promoter constructs with different 5′ deletions of ELP5 promoter in HEK293T cells. RLU, relative light units. **e** Luciferase assay of ELP5 promoter (− 450 bp to + 107 bp) activities by ELP5 promoter construct co-transfected with empty vector (EV), wild type (WT), or loss-of-function mutation (R882H) of DNMT3A constructs in HEK293T cells. **f-h** ChIP-qPCR analysis of 5mC contents in ELP5 promoter (**f**), RT-qPCR analysis of ELP5 transcription levels (**g**), and western blot analysis for ELP5 protein levels (**h**) in both NOZ and GBC-SD cells transfected with EV, WT, or R882H of DNMT3A constructs. Student’s t test for statistical analysis, *P < 0.05, **P < 0.01, ***P < 0.001

DNA methylation is catalysed by a family of enzymes called DNA methyltransferases (DNMTs), including DNMT1, DNMT3A, and DNMT3B. DNMT3A and DNMT3B are both de novo DNMTs that catalyse de novo methylation of unmethylated CpG dinucleotides, but DNMT1 mainly maintains DNA methylation during replication [22]. To identify the key DNMTs mediating the hypermethylation of the ELP5 promoter, we knocked down DNMT1, DNMT3A and DNMT3B expression in GBC cells with shRNAs. The results showed that only DNMT3A silencing restored ELP5 transcription and decreased ELP5 promoter methylation, but DNMT1 or DNMT3B silencing did not (Fig. 2a, b). ChIP-qPCR assays also showed that the content of 5mC in the ELP5 promoter was decreased by DNMT3A silencing (Fig. 2c). To determine the functional CpG islands in and the minimal essential region of the ELP5 promoter, we generated a group of promoter constructs with different 5′ deletions of the ELP5 promoter (Fig. 2d). A luciferase assay found that a 557-bp region (− 450 bp to + 107 bp, containing a CpG island around the TSS of ELP5) displayed the highest promoter activity for ELP5 transcription (Fig. 2d). Moreover, overexpression of the wild-type variant of DNMT3A inhibited the transcriptional activity of the ELP5 promoter, but the R882H loss-of-function mutation of DNMT3A did not impair the transcriptional activity of the ELP5 promoter (Fig. 2e). Coincidently, overexpression of wild-type DNMT3A in GBC cells increased the DNA methylation of the promoter, inhibited transcription, and ultimately downregulated ELP5 expression, but the R882H variant of DNMT3A did not (Fig. 2f-h). These results indicate that DNMT3A is the key DNA methyltransferase that catalyses methylation of the ELP5 promoter and mediates ELP5 transcriptional inactivation in GBC cells.

### DNA hypermethylation blocks PAX5 binding to the ELP5 promoter

**Fig. 3:**
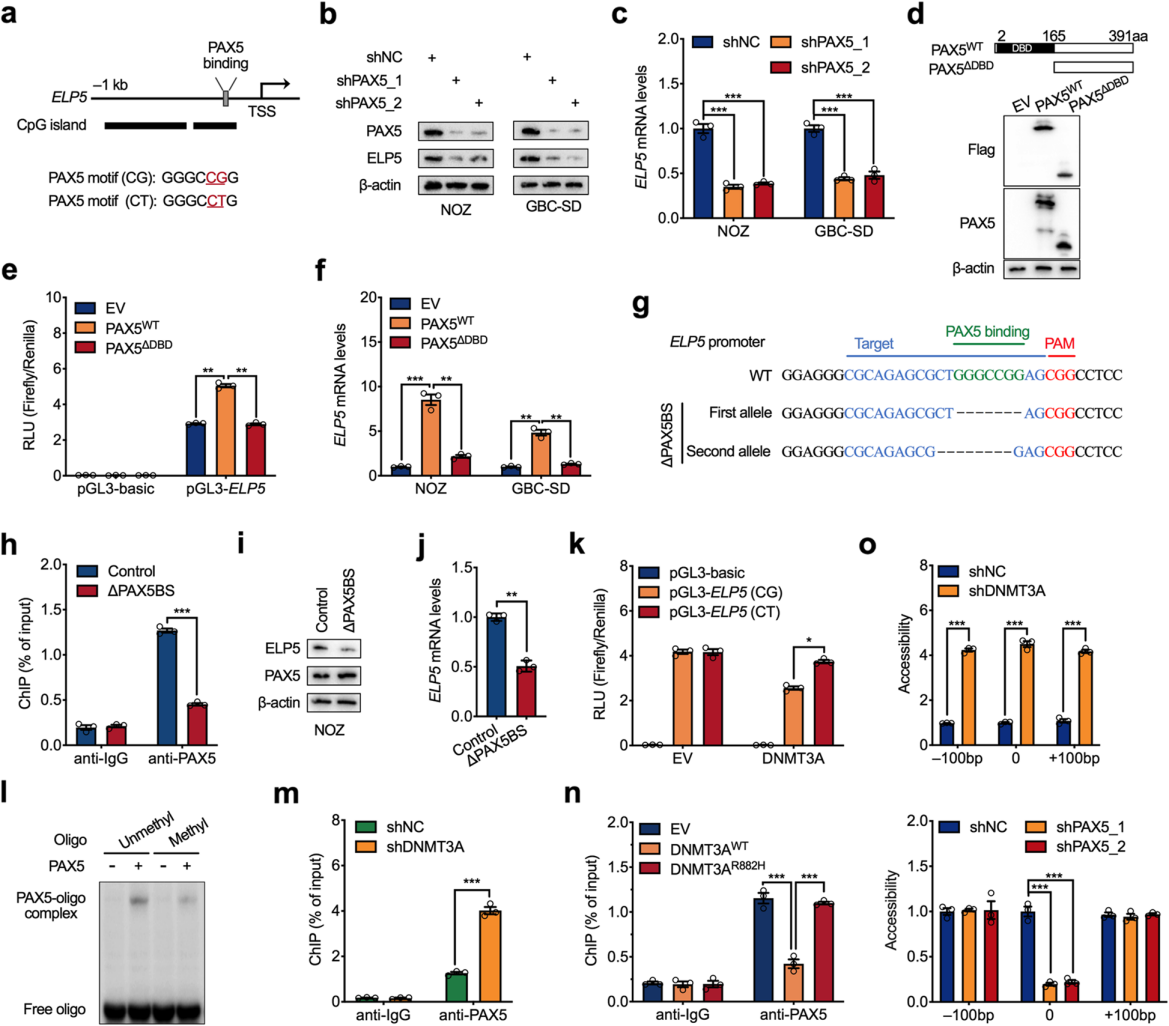
DNA hypermethylation blocks PAX5 regulation of ELP5. **a** Schematic diagram of PAX5 binding sites and nucleotide sequences on ELP5 promoter. **b,c** Western blot and RT-qPCR analysis to detect ELP5 protein levels (**b**) and transcription levels (**c**) upon PAX5 stably knockdown (shPAX5) by two independent shRNAs in NOZ and GBC-SD cells. **d** Schematic diagram of full length (WT) and DNA binding domain (DBD)-depleted fragment (△DBD) of PAX5 (top), and western blot to detect ectopically expressed WT and △DBD of PAX5 in HEK293T cells (bottom). **e** Luciferase assay of ELP5 promoter (− 450 bp to + 107 bp) activities by ELP5 promoter construct co-transfected with EV, WT, or △DBD of PAX5 construct in HEK293T cells. **f** RT-qPCR analysis of ELP5 transcription levels in NOZ and GBC-SD cells transfected with EV, WT, and △DBD of PAX5 constructs. **g** Schematic diagram showing the genomic sequences around PAX5 binding sites on ELP5 promoter in WT NOZ cell and a mutant NOZ subclone lacking the PAX5 binding site (ΔPAX5BS) generated by CRISPR/Cas9 method. **h** ChIP-qPCR analysis of PAX5 occupancy in ELP5 promoter in WT and ΔPAX5BS NOZ cells. **i, j** Western blot (**i**) and RT-qPCR analysis (**j**) to compare ELP5 expressions in WT and ΔPAX5BS NOZ cells. **k** Luciferase assay of ELP5 promoter (− 450 bp to + 107 bp) constructs containing methylation-sensitive (CG) or -negative (CT) PAX5 binding sites co-transfected with DNMT3A or EV in HEK293T cells. **l** EMSA results of PAX5 binds to the unmethylated or methylated oligonucleotides containing a PAX5 binding site in vitro. **m,n** ChIP-qPCR analysis of PAX5 occupancy in ELP5 promoter in DNMT3A stably knockdown NOZ cells (**m**) or EV, WT and R882H of DNMT3A transfected NOZ cells (**n**). **o** Chromatin accessibility is controlled by DNMT3A (top) and PAX5 (bottom) at the ELP5 promoter around PAX5 binding site in NOZ cells. The x axes indicate the distance from the PAX5 binding site. Student’s t test for statistical analysis, *P < 0.05, **P < 0.01, ***P < 0.001

To further uncover the distinct molecular mechanism by which hypermethylation represses the transcription of the ELP5 gene, we screened potential transcription factors that bind to the CpG island of the ELP5 promoter via the PROMO website [23]. A conserved PAX5 binding motif containing a CpG site was identified (Fig. 3a). Next, we investigated the regulatory effect of PAX5 on ELP5 transcription. PAX5 knockdown by two independent shRNAs decreased ELP5 transcription and protein expression in GBC cells (Fig. 3b, c). PAX5 belongs to the paired box (PAX) family of transcription factors and contains a paired box domain for DNA binding [24]. To confirm that the DNA-binding ability of PAX5 is required for binding the ELP5 promoter and activating ELP5 transcription, we generated a truncated variant of PAX5 lacking a DNA-binding domain (ΔDBD) (Fig. 3d). As confirmed by luciferase assay, the ΔDBD variant of PAX5 could not activate the activity of the ELP5 promoter, but the full-length variant of PAX5 could promote ELP5 promoter activity (Fig. 3e). Moreover, ectopic expression of the full-length variant of PAX5 in GBC cells could activate ELP5 transcription, but the ΔDBD variant could not (Fig. 3f). Further, we generated a subclone of NOZ cell lacking the PAX5 binding site (ΔPAX5BS) on the ELP5 promoter by CRISPR/Cas9-mediated approach (Fig. 3g, Fig. S1). ChIP-qPCR assay confirmed that PAX5 could not bind to the ELP5 promoter in ΔPAX5BS cells (Fig. 3h), resulted in the repressed expression of ELP5 (Fig. 3i, j). These data confirm that PAX5 could directly bind to the ELP5 promoter and mediate ELP5 transcriptional activation.

To determine the role of DNA methylation in PAX5-induced activation of ELP5, we introduced a site-specific mutation within the PAX5 binding motif (CG to CT), which caused the promoter construct to lack methylation ability (Fig. 3a). A luciferase assay confirmed that this methylation-disabled variant of the PAX5 binding motif interfered with the ability of DNMT3A to methylate and repress ELP5 promoter activity (Fig. 3k) and interfered with the in vitro methylation of the ELP5 promoter (Fig. S2a, b). After in vitro methylation, the ability of PAX5 to transactivate the ELP5 promoter was significantly reduced in the wild-type variant of the PAX5 binding motif but was not affected in the methylation-disabled variant (Fig. S2c). To determine whether the DNA methylation on PAX5 binding site directly perturbed PAX5-DNA interaction, we utilized the electrophoretic mobility shift assay (EMSA) and the result showed PAX5 less binding to the methylated oligo compared to the unmethylated oligo in vitro (Fig. 3l). ChIP-qPCR assays also confirmed that PAX5 occupancy in the ELP5 promoter was significantly abolished by DNMT3A-mediated methylation in vivo (Fig. 3m, n). These data suggest that DNA methylation could directly interfere PAX5 binding to its targeted chromatin loci.

In addition to directly blocking transcription factors’ binding, DNA methylation within CpG dinucleotides has the potential to restrict the accessibility of chromatin structure to form the transcriptionally repressive chromatin environments [25, 26]. Highly compressed chromatin structure could also prevent transcription factors access to regulatory elements. We then performed a chromatin accessibility assay to determine the effect of DNA methylation or PAX5 protein on chromatin-remodeling activity at the locus of ELP5 promoter using micrococcal nuclease (MNase)-mediated method. As anticipated, the loss of DNMT3A induced a broad increased accessibility, including PAX5 binding region (Fig. 3o). Besides, knockdown of PAX5 could only decrease the chromatin accessibility at PAX5 binding region, but not at more distant loci (Fig. 3o), carried out a conclusion that PAX5-mediated the opening of the chromatin accessibility is specific for ELP5 transcription. These data provide the second mechanism for explaining DNA methylation acts in PAX5-DNA interactions is that DNA methylation inhibits the opening of chromatin conformation to prevent the accessibility of PAX5 to the targeted chromatin loci.

To validate PAX5-activated or DNMT3A-inhibited ELP5 expression determine the gemcitabine sensitivity in GBC cells, we performed gemcitabine sensitivity assays in GBC cells with ΔPAX5BS or DNMT3A deletion. The results showed that NOZ cells with ΔPAX5BS exhibited more resistant to gemcitabine (Fig. S3a), but deletion of DNMT3A sensitized NOZ and GBC-SD cells to gemcitabine (Fig. S3b). These data confirm that ELP5 expression regulated by PAX5 or DNMT3A generated opposite effects on gemcitabine sensitivity.

Taken together, these results indicate that DNA methylation in PAX5 binding motif could direct interfere PAX5-DNA interaction and also decrease the chromatin accessibility to restrict PAX5 binding to chromatin, which draw a conclusion that PAX5-mediated activation of ELP5 transcription is controlled by DNA methylation and is methylation-dependent.

### A DNA demethylating agent activates ELP5 expression

**Fig. 4:**
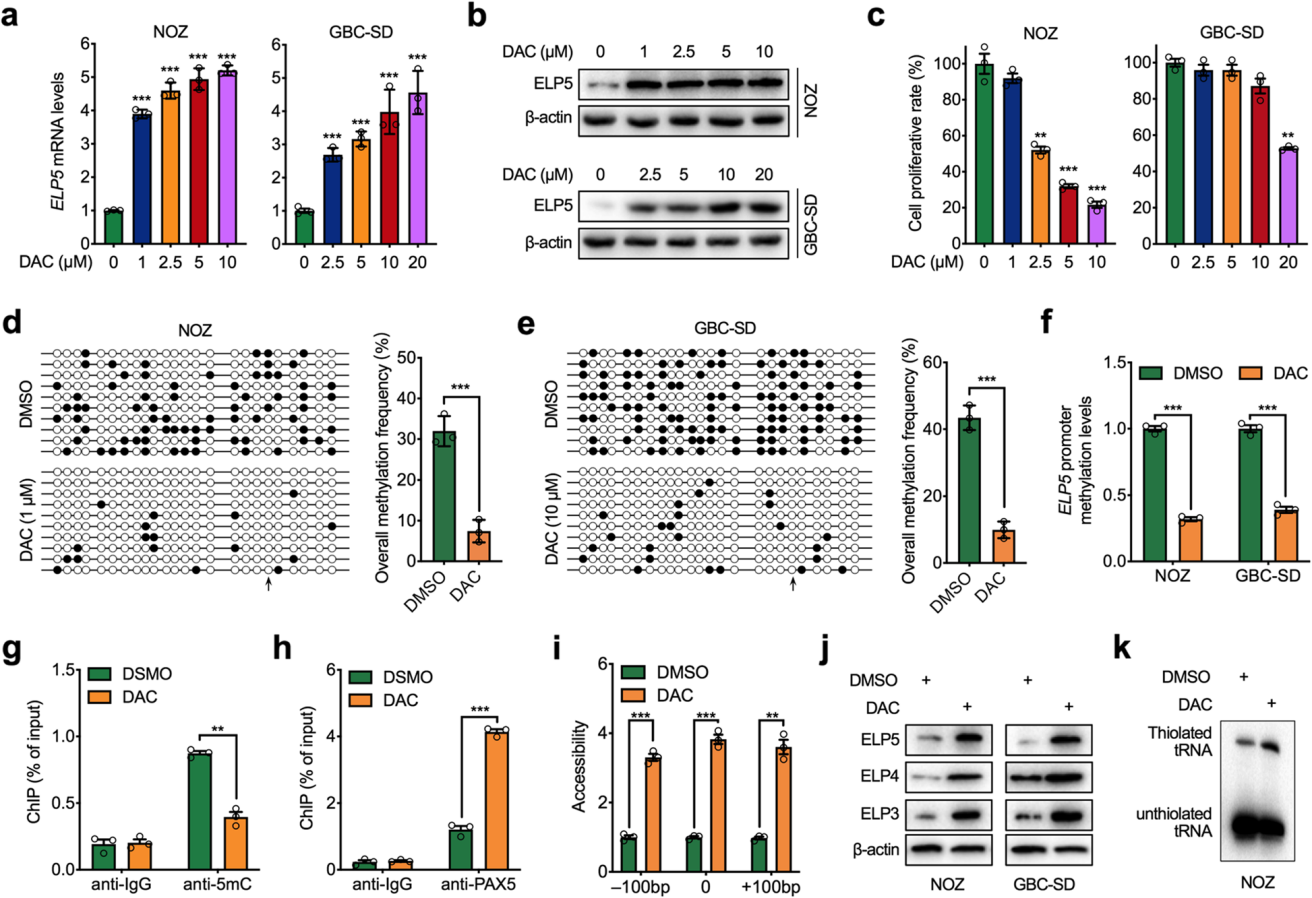
Decitabine activates ELP5 expression. **a,b** RT-qPCR and western blot analysis to detect ELP5 transcription levels (**a**) and protein levels (**b**) in NOZ and GBC-SD cells treated with decitabine (DAC) at the indicated dosage for 72 h. **c** Cell viability analysis for NOZ and GBC-SD cells treated with DAC at the indicated dosage for 72 h. **d,e** Bisulfite sequencing PCR (BSP) assays for the methylation of ELP5 promoter in NOZ and GBC-SD cells treated with DAC (1 μM for NOZ (**d**) and10 μM for GBC-SD (**e**)) or DMSO (vehicle) for 72 h. ○ indicates unmethylated CpG sites and ● indicates methylated CpG sites (left panel). The bar graphs depict the ELP5 promoter methylation rates (right panel). **f** MS-qPCR analysis of ELP5 promoter methylation levels in NOZ and GBC-SD cells treated with DAC (1 μM for NOZ and 10 μM for GBC-SD) or DMSO for 72 h. **g,h** ChIP-qPCR analysis of 5mC content (**g**) and PAX5 occupancy (**h**) in ELP5 promoter in NOZ cells treated with 1 μM DAC or DMSO for 72 h. **i** Chromatin accessibility of ELP5 promoter in NOZ cells treated with 1 μM DAC or DMSO for 72 h. The x axes indicate the distance from the PAX5 binding site. **j** Western blot for ELP5 and another two subunits of elongator complex (ELP4 and ELP3) protein levels in NOZ and GBC-SD cells treated with DAC (1 μM for NOZ and 10 μM for GBC-SD) or DMSO for 72 h. **k** Northern blot for the thiolated tRNA abundance in NOZ cells treated with 1 μM DAC or DMSO for 72 h. Student’s t test for statistical analysis, *P < 0.05, **P < 0.01, ***P < 0.001

DAC, a DNA demethylating agent that inhibits DNMTs-mediated DNA methylation in cancer cells, has been widely used as a promising agent to activate hypermethylated tumour suppressor gene expression [27]. Thus, we explored the demethylating effect of DAC on the ELP5 promoter. Upon DAC treatment, ELP5 transcription was activated, and protein accumulated in a dose-dependent manner in GBC cells (Fig. 4a, b). Moreover, DAC induced cell growth arrest in a dose-dependent manner (Fig. 4c). Next, we treated the GBC cells with a minimal dose of DAC that could activate ELP5 transcription but had minor cell growth arrest effects. Bisulfite sequencing PCR (BSP) assays confirmed that DAC could demethylate CpG islands and completely abolish the hypermethylation of the CpG site located in PAX5 binding motif within the ELP5 promoter (Fig. 4d, e). Further verification by MS-qPCR showed that DAC treatment decreased the methylation level of the ELP5 promoter (Fig. 4f). Coincidently, the content of 5mC was reduced, but the occupancy of PAX5 was enriched in the ELP5 promoter upon DAC treatment in GBC cells (Fig. 4g, h). As anticipated, DAC treatment could increase the accessibility of ELP5 promoter loci (Fig. 4i).

ELP5 mainly functions as an organizer to maintain the integrity and stability of the elongator complex and enhance the U_34_ tRNA-catalysing ability of the elongator complex [5, 6, 28]. To confirm that the enhanced expression of ELP5 by DAC treatment was functional, we analysed the expression of other subunits of the elongator complex and the content of modified U34 tRNA upon DAC treatment. The results showed that DAC induced ELP5 protein expression together with the increased expression of other subunits of the elongator complex in GBC cells (Fig. 4j), accompanied by the increased content of modified U34 tRNA (Fig. 4k). In summary, we conclude that the DNA demethylating agent DAC could demethylate the ELP5 promoter, transactivate PAX5-mediated ELP5 transcription, and increase the U_34_ tRNA-catalysing ability of the elongator complex.

### A DNA demethylating agent sensitizes GBC cells to gemcitabine via ELP5 activation

**Fig. 5:**
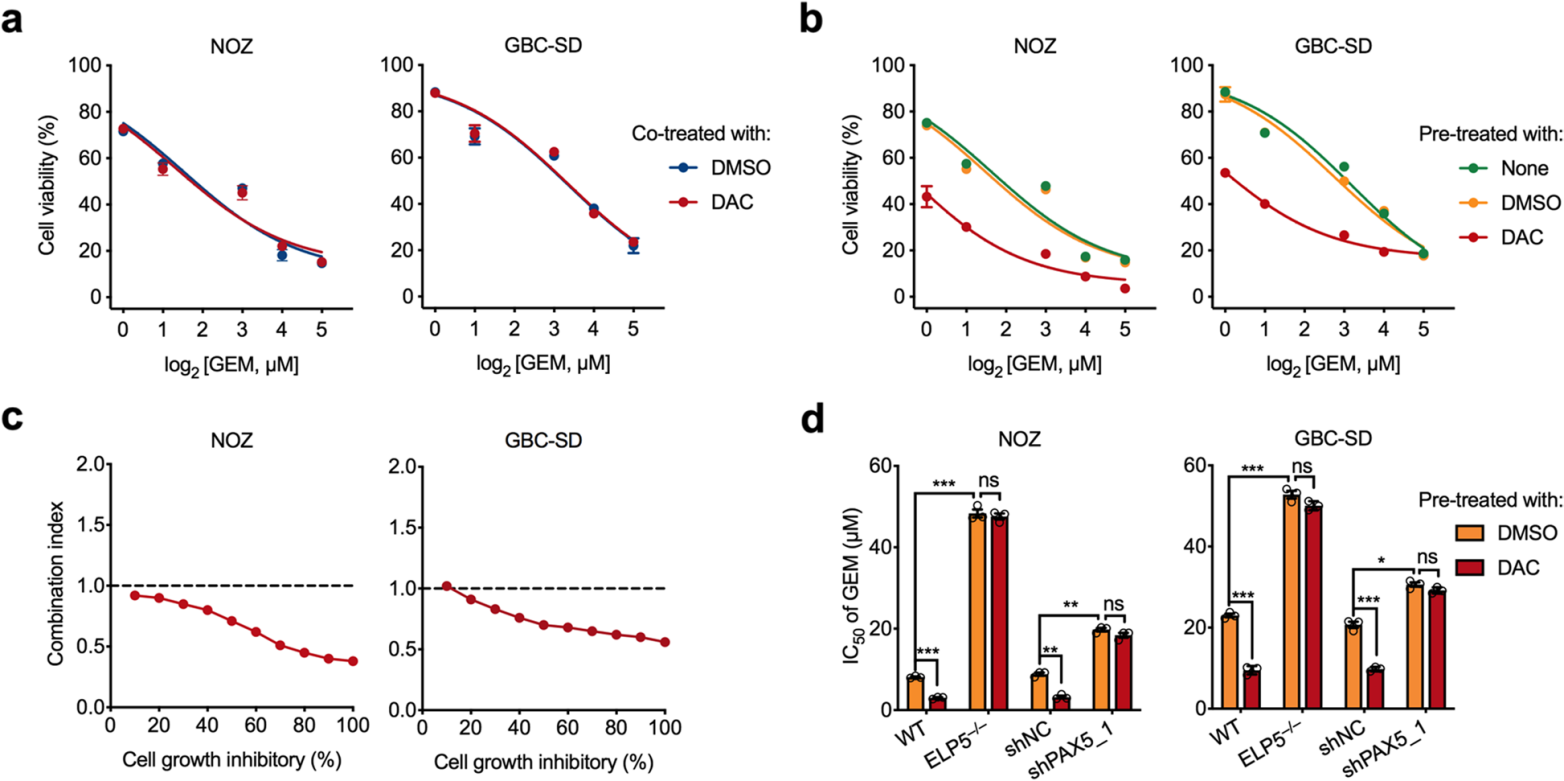
DAC sensitizes GBC cells to gemcitabine via ELP5 activation. **a** Cell viability analysis for NOZ and GBC-SD cells treated with gemcitabine (GEM) at the indicated dosage synchronously combined with DAC (1 μM for NOZ and 10 μM for GBC-SD) or DMSO (vehicle) for 72 h. **b** NOZ and GBC-SD cells were pre-treated with DAC (1 μM for NOZ and 10 μM for GBC-SD), vehicle, or none any chemicals for 72 h. After that, these cells were sequentially treated with GEM at the indicated dosage for next 72 h. Cell viability assays were used to compare the gemcitabine sensitivities in different chemicals or no chemical pre-treated cells. **c** Combination index-fraction affected plots of 72 h DAC pre-treated and 72 h GEM sequentially treated in NOZ and GBC-SD cells. Plots were generated using the CompuSyn software. Combination index (CI) < 1, CI = 1, and CI > 1 indicate synergism, additive effect, and antagonism, respectively. Smaller CI value indicates stronger synergism. **d** Cell viability analysis for ELP5-depleted or PAX5-silenced NOZ and GBC-SD cells treated with DAC and GEM sequential combinations. Student’s t test for statistical analysis, ns, not significant, *P < 0.05, **P < 0.01, ***P < 0.001

Gemcitabine is a nucleoside analogue of deoxycytidine and the most commonly used agent for the treatment of patients with GBC [29], and the status of ELP5 determines GBC cell resistance to gemcitabine [5]. On the basis of the pivotal role of DNA methylation-mediated ELP5 transcription repression in GBC, we examined whether reactivation of ELP5 by DAC could sensitize GBC cells to gemcitabine. Interestingly, co-treatment with a low dose of DAC did not sensitize GBC cells to gemcitabine (Fig. 5a), but GBC cells pre-treated with a low dose of DAC for 72 h showed increased gemcitabine sensitivity (Fig. 5b). As evaluated by the Chou-Talalay method [30], the sequential combination of a low dose of DAC and gemcitabine exhibited synergistic effects, with the combination index consistently below 1.0 in GBC cells (Fig. 5c). However, endogenous ELP5 depletion or PAX5 silencing markedly abolished GBC cell sensitization to gemcitabine by DAC pre-treatment (Fig. 5d), which suggested that the DAC-induced sensitization to gemcitabine cytotoxic effects partially required ELP5 and PAX5 expression. Together, these results prove the indispensable role of ELP5 in the synergistic effects of sequential DAC and gemcitabine combination therapy.

**Fig. 6:**
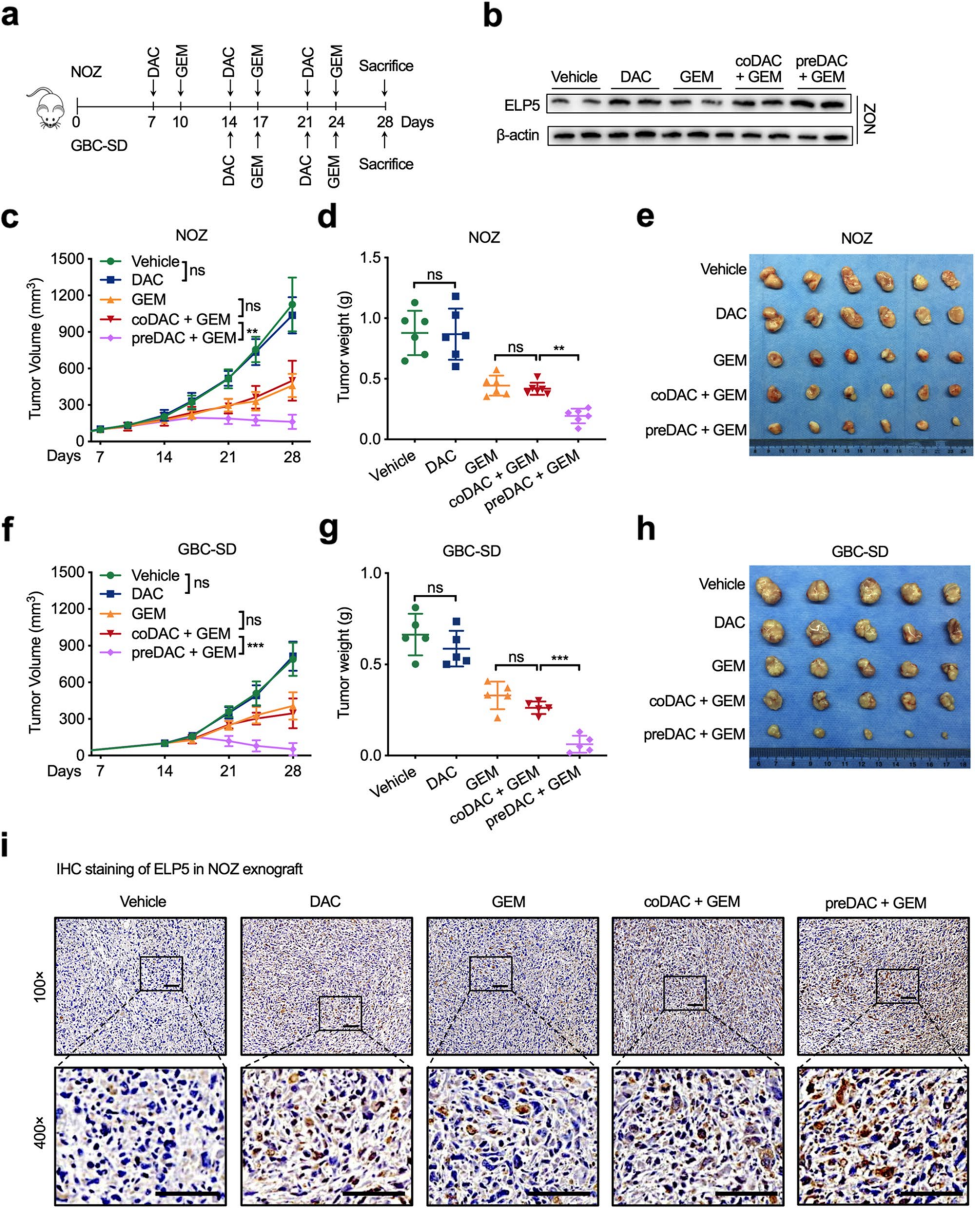
DAC sensitizes GBC cells to gemcitabine in vivo. **a** Experiment timeline and dosing schedule for xenograft models. **b** Western blot for ELP5 protein levels in NOZ xenograft tumours undergoing different treatments. **c** Tumour growth curves in NOZ xenograft models. **d** Statistical analysis of tumour weight in NOZ xenograft tumours after scarification. **e** Representative images of NOZ xenograft tumours after scarification. **f** Tumour growth curves in GBC-SD xenograft models. **g** Statistical analysis of tumour weight in GBC-SD xenograft tumours after scarification. **h** Representative images of GBC-SD xenograft tumours after scarification. **i** Representative immunohistochemistry of ELP5 proteins in paraffin-fixed NOZ xenograft tissues. Scale bar = 100 μm. One-way ANOVA test for statistical analysis, ns, not significant, **P < 0.01, ***P < 0.001

The synergism in the sequential drug combination of DAC and gemcitabine was also validated in GBC cell line xenograft models (Fig. 6a). ELP5 expression in xenograft tumours was significantly induced under DAC pre-treatment (Fig. 6b). With regard to tumour growth, no differences were observed in tumour volume increase and tumour weight decrease between the low-dose DAC co-treated with gemcitabine group and the single-agent gemcitabine treatment group, but the sequential combination of DAC and gemcitabine not only remarkably delayed tumour volume increase but also resulted in tumour weight decrease compared to the low-dose DAC co-treated with gemcitabine treatment and the single-agent gemcitabine treatment (Fig. 6c-e for the NOZ xenograft, Fig. 6f-h for the GBC-SD xenograft). Immunohistochemistry also confirmed that sequential combination of DAC and gemcitabine obviously induced ELP5 expression in xenograft models (Fig. 6i, Fig. S4a). Since we utilized a low dose of DAC in the xenograft models, no nephrotoxicity or liver toxicity was observed in the single-agent groups or combination groups by histological analysis of kidney and liver sections (Fig. S4b), which demonstrated that low-dose DAC pre-treatment in gemcitabine therapy is safe and effective.

Collectively, these results confirm that transcriptional activation of ELP5 by DAC sensitizes GBC cells to gemcitabine therapy both in vitro and in vivo xenografts, and the sequential combination of DAC and gemcitabine exhibit enhanced synergistic effects in GBC therapy.

### The levels of ELP5, PAX5, and DNMT3A are correlated in GBC tissues

**Fig. 7:**
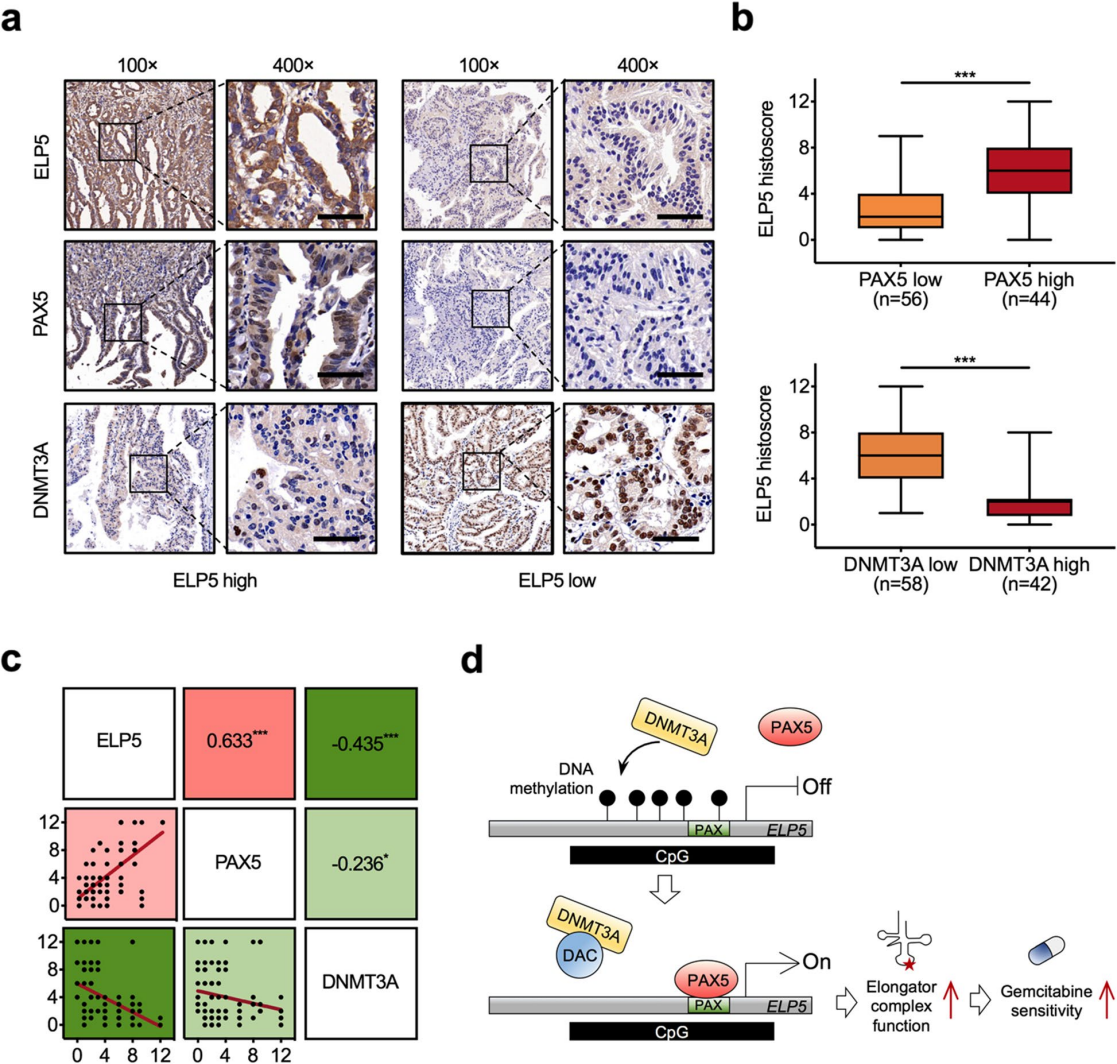
ELP5, PAX5, and DNMT3A correlate and are relevant in GBC. **a** Representative immunohistochemistry of ELP5 (top), PAX5 (middle), and DNMT3A (bottom) proteins in GBC tissues from patients with high or low ELP5 expression. Scale bar = 50 μm. **b** Statistical analysis of the histoscore of ELP5 proteins in GBC tissues from patients with high or low PAX5 expression (top), and DNMT3A expression (bottom). Student’s t test for statistical analysis, ***P < 0.001. **c** Correlations among ELP5, PAX5, and DNMT3A protein levels in GBC tissues. Pearson correlation coefficient for statistical analysis, *P < 0.05, ***P < 0.001. **d** Schematic diagram for the epigenetic activation of ELP5 by DAC enhances the gemcitabine cytotoxic effects in GBC

To investigate the expression correlations of ELP5, PAX5, and DNMT3A in GBC tissues, we quantified the expression of each protein by immunohistochemistry. We found that low expression of ELP5 was more likely to be detected in GBC tissues with low PAX5 or high DNMT3A expression (Fig. 7a, b). The protein expression of ELP5 in GBC tissues was positively correlated with PAX5 expression but negatively correlated with DNMT3A expression (Fig. 7c). Besides, the expression of PAX5 was also negatively correlated with DNMT3A (Fig.7c).

On the basis of these findings, we conclude that the occupancy of PAX5 at the ELP5 promoter is controlled by DNA methylation and that hypermethylation prevents PAX5 binding and represses ELP5 transcription. In contrast, DNA demethylating agents such as DAC can remove the methylated CpGs within the ELP5 promoter, allowing PAX5 to rebind to the ELP5 promoter and reactivate ELP5 transcription, ultimately resulting in GBC cell sensitization to gemcitabine (Fig. 7d).

## Discussion

Gemcitabine-based chemotherapy has been widely used in various types of cancer, including GBC. Although GBC patients respond well to surgical resection and benefit from chemotherapy, relapse with chemoresistant cancer usually occurs and patients succumb to disease. Due to the lack of effective targeted therapy and unknown immunotherapy efficacy, conventional chemotherapy remains the first-line treatment for GBC patients. Identifying drug resistance targets and finding targeted reagents have become important strategies for improving the effectiveness and overcoming the resistance of conventional chemotherapy.

Genetic and epigenetic alterations in GBC have been extensively reported [31–33]. Recent studies have shown that hypermethylation induces silencing of tumour suppressor genes in GBC, and the progression of GBC is accompanied by an increased frequency of methylation [34, 35]. However, the potential role of DNA methylation in GBC chemoresistance is poorly understood. Previously, we showed that loss of ELP5 determines gemcitabine resistance in GBC [5], but the distinct mechanism that mediates ELP5 silencing in GBC remains unclear. Through a combination of genetic, epigenetic, transcriptional, and biological studies, we demonstrated that ELP5 transcription is repressed in GBC patients with hypermethylation of the ELP5 promoter.

In GBC, hypermethylation is the dominant status in differentially methylated sites [34]. The majority of hypermethylated sites are localized to the proximal promoter region [containing the TSS and 5′ untranslated region (5′-UTR)] and the first exon. A large number of CpG dinucleotide repeats form CpG islands, which are typically common near the TSS and result in stable silencing of gene expression. Two CpG islands located in the ELP5 promoter region were predicted, and a 271-bp region containing 21 CpG sites upstream the TSS was identified as a major methylated region modulating the transcriptional activity of ELP5, indicating that the ELP5 promoter in GBC is characterized by a hypermethylated CpG island.

DNA methylation is thought to regulate transcription both directly and indirectly. CpG methylation was found to directly repress transcription by preventing transcription factors from binding to their recognition motifs [36]. Here, we identified DNMT3A as the main DNA methyltransferase generating methylation of the ELP5 promoter and found that hypermethylation of the ELP5 promoter prevents the binding of the transcription factor PAX5 and represses PAX5-induced ELP5 transcription, highlighting a tumour suppressor role of PAX5 in GBC. PAX5 is a member of the PAX transcription factor family, which plays a crucial and indispensable role in various developmental processes [37]. Lack or inactivation of PAX5 results in tumour progression and promotes a malignant state [38]. PAX5 participates in a multitude of oncogenic events in human malignancies, but the most common alteration is partial inactivation of this gene [39–41]. As a transcription factor, the primary function of PAX5 is binding to the upstream regulatory element of target genes in the promoter or enhancer regions to activate transcription. We identified the binding motif of PAX5 in the CpG island of the ELP5 promoter and found that the methylation of CpG dinucleotides within the PAX5 binding motif could block PAX5 binding and that PAX5-activated ELP5 transcription was repressed by DNMT3A-catalysed hypermethylation of the ELP5 promoter. Mechanistically, CpG dinucleotides methylation in PAX5 binding motif directly interferes PAX5-DNA interaction, and also decrease the chromatin accessibility to restrict PAX5 binding to ELP5 promoter. Thus, we conclude that PAX5 exhibits a methylation-sensitive DNA binding ability, and methylation of the ELP5 promoter prevents PAX5 activation of ELP5 in an epigenetic approach. Our data may explain why GBC patients with low expression of ELP5 experience gemcitabine resistance.

It’s surprising that the expression of PAX5 in GBC tissues was negatively correlated with DNMT3A. It’s well reported that PAX5 promoter is hypermethylated in various cancer types, including hepatocellular carcinoma, gastric cancer and esophageal cancer, resulted in the down-regulated expression of PAX5 [42–44]. But it’s still unknown which DNMT catalyses the hypermethylation of PAX5. As showed in our result, DNMT3A may be the key DNMT for PAX5 hypermethylation that develops a hypothesis that DNA methylation might repress PAX5 transcription, resulting in insufficient PAX5 binding to its target genome loci. Besides, we found knockdown of PAX5 could not maintain the enhanced gemcitabine sensitivity by DAC, suggesting that DAC might act partially through PAX5 expression to enhance the sensitivity of gemcitabine, for the reason that DAC could also restore PAX5 expression by DAC-mediated demethylation [42]. These two speculations, which require further demonstration, may provide a better understanding of the relationship between PAX5 methylation and its biological function in GBC.

The central question addressed in this study is whether reactivation of the elongator complex by a DNA demethylating agent can rescue gemcitabine sensitivity in GBC. Gemcitabine is a nucleoside analogue of deoxycytidine that induces DNA damage and inhibits DNA replication [45]. Mechanistically, gemcitabine-induced DNA damage activates the p53-initiated classical intrinsic pathway of apoptosis, thereby arresting tumour growth and triggering cell death [46]. Thus, insufficient DNA damage cascades can cause gemcitabine resistance in tumour cells. Our prior study uncovered that loss of ELP5 impairs the integrity and stability of the elongator complex to disrupt IRES-driven translation of p53 and minimize the potent p53-dependent apoptosis induced by gemcitabine in GBC [5]. Inadequate expression of ELP5 and an impaired elongator complex result in gemcitabine therapy failure. Under DAC treatment, hypermethylation of the ELP5 promoter was eliminated, and the expression of ELP5 was restored, followed by enhancement of the function of the elongator complex and the abundance of modified U34 tRNAs in GBC cells. The sequential combination of DAC and gemcitabine therapy was also confirmed as an effective and secure strategy to sensitize GBC cells to gemcitabine both in vitro and in vivo.

In addition to ELP5, the expression of ELP3, ELP4 and other subunits of the elongator complex are also controlled by DNA methylation [47, 48]. In present study, we found DAC could reactive ELP5 transcription followed by the increased protein accumulation of other subunits of the elongator complex. It remains unknown that whether DAC controls ELP5 expression to increase the stability of elongator complex, or directly activates the transcription of other subunits of the elongator complex. This question deserves to be further explored and may provide a more comprehensive view on DAC effect for the enhanced function of the elongator complex.

## Conclusions

In summary, we found for the first time that ELP5 exhibits DNA methylation-dependent, PAX5-driven transcription in GBC and that the DNA demethylating agent DAC can enhance the U34 modification function of the elongator complex via ELP5 reactivation (Fig. 7d). Our work provides a novel therapeutic strategy in patients with GBC: sequential combination treatment with DAC and gemcitabine, which could be a promising treatment option that sensitizes GBC cells to gemcitabine by epigenetically activating the elongator complex.

## Supplementary Information

**Additional file 1: Fig. S1.** Validation of the deletion of PAX5 binding site in ELP5 promoter. Sanger sequencing results show the natural sequences around PAX5 binding site in ELP5 promoter in wild type NOZ cells (top) and the mutated sequences of both alleles in NOZ cells lacking the PAX5 binding site (ΔPAX5BS). **Fig. S2.** In vitro methylation of ELP5 promoter shows low transcription activity. a Validation of in vitro methylation efficiency of ELP5 promoter constructs treated by M.SssI or mock treated followed by the methylation-sensitive restriction enzyme HpaII digestion. b Luciferase assay of in vitro methylated ELP5 promoter constructs contain methylation-sensitive (CG) or negative (CT) PAX5 binding sites in HEK293T cells. **c** Luciferase assay of in vitro methylated ELP5 promoter constructs contain methylation-sensitive (CG) or negative (CT) PAX5 binding sites co-transfected with PAX5 in HEK293T cells. Student’s t test for statistical analysis, **P < 0.01, ***P < 0.001. **Fig. S3.** The effect of PAX5-DNA binding and DNMT3A in gemcitabine sensitivity. a Cell viability analysis for NOZ cells lacking the PAX5 binding site (ΔPAX5BS) and control wild type (WT) cells treated with gemcitabine (GEM) at the indicated dosage for 72 h. b Cell viability analysis for DNMT3A knockdown (shDNMT3A) and control (shNC) NOZ and GBC-SD cells treated with GEM at the indicated dosage for 72 h. **Fig. S4.** Neither nephrotoxicity nor hepatotoxicity in mice undergoing different treatments. a Representative immunohistochemistry of ELP5 proteins in paraffin-fixed GBC-SD xenograft tissues. Scale bar = 100 μm. b H&E staining in kidney (top) and liver (bottom) in mice undergoing different treatments. Scale bar = 200 μm.

## Data Availability

The datasets used and/or analyzed during the current study are available from the corresponding author on reasonable request.

## Abbreviations

5mC: 5-methylcytosine
BSP: bisulfite sequencing PCR
EMSA: electrophoretic mobility shift assay
DBD: DNA binding domain
ChIP: chromatin immunoprecipitation
cm^5^U: 5-carbamoylmethyluridine
DAC: decitabine
ELP5: the elongator complex subunit 5
DNMTs: DNA methytransferases
GEM: gemcitabine
GBC: gallbladder cancer
HEK293T: human embryonic kidney 293 T
IRES: the internal ribosomal entry site
MS-qPCR: methylation-specific quantitative PCR
PAX: the paired box
shRNA: short hairpin RNA
TSS: the transcription start site
UTR: untranslated region
U34: uridine 34
