# Supplementary material for: Epigenetic activation of the elongator complex sensitizes gallbladder cancer to gemcitabine therapy
Source: J Exp Clin Cancer Res. 2021 Nov 25;40:373. doi: 10.1186/s13046-021-02186-0 (PMC8613969; doi:10.1186/s13046-021-02186-0)
Supplement: Supplementary file 1 — Additional file 1: Fig. S1. Validation of the deletion of PAX5 binding site in ELP5 promoter. Sanger sequencing results show the natural sequences around PAX5 binding site in ELP5 promoter in wild type NOZ cells (top) and the mutated sequences of both alleles in NOZ cells lacking the PAX5 binding site (ΔPAX5BS). Fig. S2. In vitro methylation of ELP5 promoter shows low transcription activity. a Validation of in vitro methylation efficiency of ELP5 promoter constructs treated by M.SssI or mock treated followed by the methylation-sensitive restriction enzyme HpaII digestion. b Luciferase assay of in vitro methylated ELP5 promoter constructs contain methylation-sensitive (CG) or negative (CT) PAX5 binding sites in HEK293T cells. c Luciferase assay of in vitro methylated ELP5 promoter constructs contain methylation-sensitive (CG) or negative (CT) PAX5 binding sites co-transfected with PAX5 in HEK293T cells. Student’s t test for statistical analysis, **P < 0.01, ***P < 0.001. Fig. S3. The effect of PAX5-DNA binding and DNMT3A in gemcitabine sensitivity. a Cell viability analysis for NOZ cells lacking the PAX5 binding site (ΔPAX5BS) and control wild type (WT) cells treated with gemcitabine (GEM) at the indicated dosage for 72 h. b Cell viability analysis for DNMT3A knockdown (shDNMT3A) and control (shNC) NOZ and GBC-SD cells treated with GEM at the indicated dosage for 72 h. Fig. S4. Neither nephrotoxicity nor hepatotoxicity in mice undergoing different treatments. a Representative immunohistochemistry of ELP5 proteins in paraffin-fixed GBC-SD xenograft tissues. Scale bar = 100 μm. b H&E staining in kidney (top) and liver (bottom) in mice undergoing different treatments. Scale bar = 200 μm. [file 13046_2021_2186_MOESM1_ESM.pdf]

Epigenetic activation of the elongator complex sensitizes gallbladder cancer to gemcitabine therapy

Xu et al.

Additional file:

Fig. S1 Validation of the deletion of PAX5 binding site in ELP5 promoter.

Fig. S2 In vitro methylation of ELP5 promoter shows low transcription activity.

Fig. S3 The effect of PAX5-DNA binding and DNMT3A in gemcitabine sensitivity.

Fig. S4 Neither nephrotoxicity nor hepatotoxicity in mice undergoing different treatments.

Fig. S1

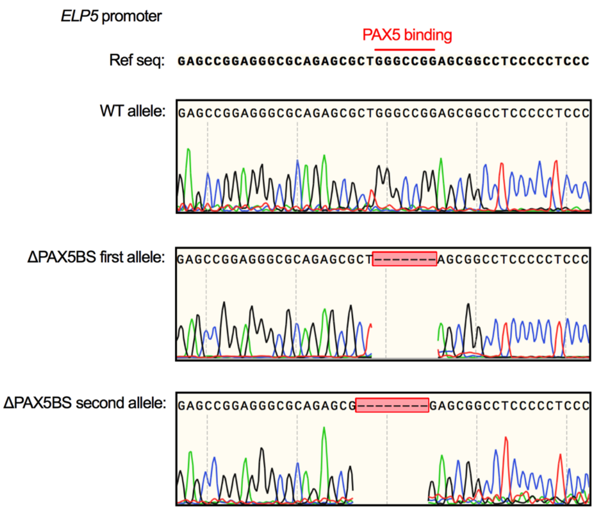

Fig. S1 Validation of the deletion of PAX5 binding site in ELP5 promoter. Sanger sequencing results show the natural sequences around PAX5 binding site in ELP5 promoter in wild type NOZ cells (top) and the mutated sequences of both alleles in NOZ cells lacking the PAX5 binding site (ΔPAX5BS).

Fig. S2

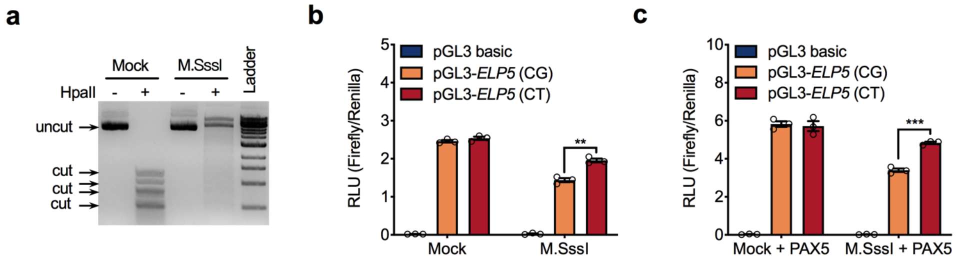

Fig. S2 In vitro methylation of ELP5 promoter shows low transcription activity. **a** Validation of in vitro methylation efficiency of ELP5 promoter constructs treated by M.SssI or mock treated followed by the methylation-sensitive restriction enzyme HpaII digestion. **b** Luciferase assay of in vitro methylated ELP5 promoter constructs contain methylation-sensitive (CG) or negative (CT) PAX5 binding sites in HEK293T cells. **c** Luciferase assay of in vitro methylated ELP5 promoter constructs contain methylation-sensitive (CG) or negative (CT) PAX5 binding sites co-transfected with PAX5 in HEK293T cells. Student's t test for statistical analysis, **P<0.01, ***P<0.001.

Fig. S3

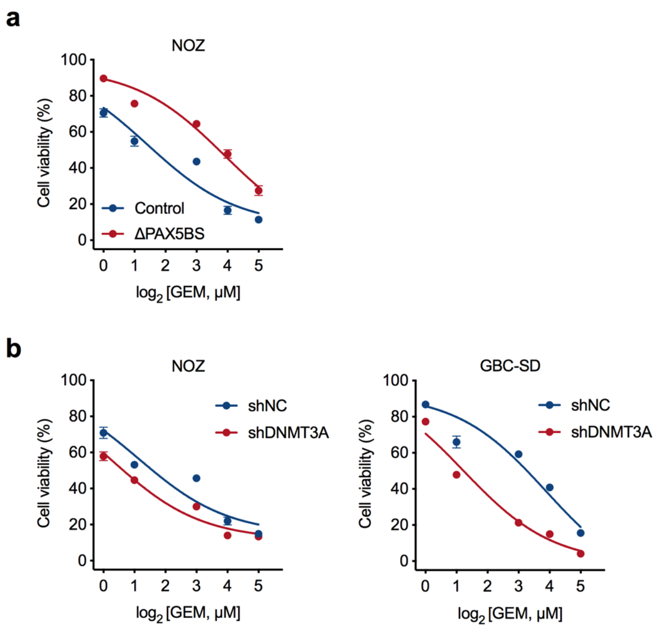

Fig. S3 The effect of PAX5-DNA binding and DNMT3A in gemcitabine sensitivity. **a** Cell viability analysis for NOZ cells lacking the PAX5 binding site (ΔPAX5BS) and control wild type (WT) cells treated with gemcitabine (GEM) at the indicated dosage for 72 hr. **b** Cell viability analysis for DNMT3A knockdown (shDNMT3A) and control (shNC) NOZ and GBC-SD cells treated with GEM at the indicated dosage for 72 hr.

Fig. S4

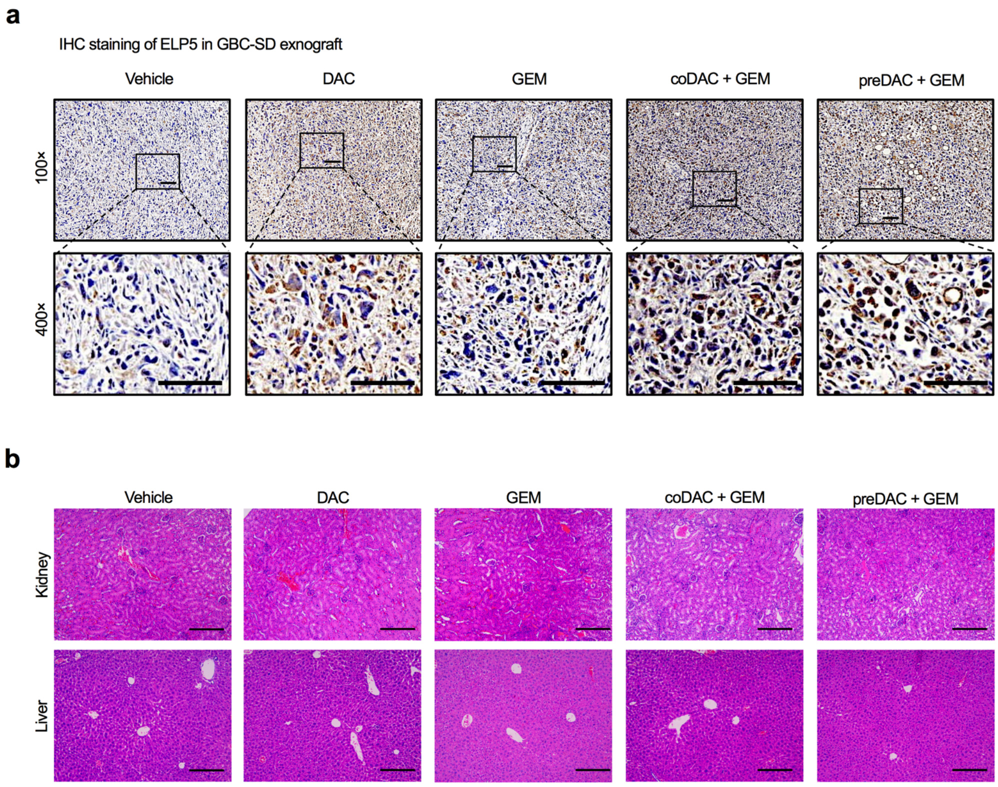

Fig. S4 Neither nephrotoxicity nor hepatotoxicity in mice undergoing different treatments. **a** Representative immunohistochemistry of ELP5 proteins in paraffin-fixed GBC-SD xenograft tissues. Scale bar = 100 μm. **b** H&E staining in kidney (top) and liver (bottom) in mice undergoing different treatments. Scale bar = 200 μm.
